# Indirect Radioiodination of DARPin G3 Using N-succinimidyl-*Para*-Iodobenzoate Improves the Contrast of HER2 Molecular Imaging

**DOI:** 10.3390/ijms20123047

**Published:** 2019-06-21

**Authors:** Anzhelika Vorobyeva, Alexey Schulga, Sara S. Rinne, Tyran Günther, Anna Orlova, Sergey Deyev, Vladimir Tolmachev

**Affiliations:** 1 Department of Immunology, Genetics and Pathology, Uppsala University, 75185 Uppsala, Sweden; anzhelika.vorobyeva@igp.uu.se (A.V.); tyrangunther@gmail.com (T.G.); 2 Molecular Immunology Laboratory, Shemyakin & Ovchinnikov Institute of Bioorganic Chemistry, Russian Academy of Sciences, 117997 Moscow, Russia; schulga@gmail.com (A.S.); deyev@ibch.ru (S.D.); 3 Department of Medicinal Chemistry, Uppsala University, 75123 Uppsala, Sweden; sara.rinne@pet.medchem.uu.se (S.S.R.); anna.orlova@ilk.uu.se (A.O.); 4 Science for Life Laboratory, Uppsala University, 75123 Uppsala, Sweden; 5 Bio-Nanophotonic Lab, Institute of Engineering Physics for Biomedicine (PhysBio), National Research Nuclear University ‘MEPhI’, 115409 Moscow, Russia; 6 Nuclear Medicine Department, Cancer Research Institute, Tomsk National Research Medical Center Russian Academy of Sciences, 634050 Tomsk, Russia

**Keywords:** DARPin, HER2, imaging, radionuclide, iodine, radioiodination

## Abstract

Radionuclide molecular imaging of human epidermal growth factor receptor 2 (HER2) in breast and gastroesophageal cancer might be used to stratify patients for HER2-targeted therapy as well as monitor treatment response and disease progression. Designed ankyrin repeat proteins (DARPins) are small engineered scaffold proteins with favorable properties for molecular imaging. Herein we compared two methods for labeling the anti-HER2 DARPin (HE)_3_-G3, direct and indirect radioiodination. We hypothesized that the use of N-succinimidyl-*para*-iodobenzoate (SPIB) for radioiodination would facilitate the clearance of radiometabolites and improve the contrast of imaging. Both radiolabeled (HE)_3_-G3 variants preserved their binding specificity and high affinity to HER2-expressing cells. The specificity of tumor targeting in vivo was also demonstrated. A biodistribution comparison of [^125^I]I-(HE)_3_-G3 and [^125^I]I-PIB-(HE)_3_-G3, in mice bearing HER2 expressing SKOV3 xenografts, showed rapid clearance of [^125^I]I-PIB-(HE)_3_-G3 from normal organs and tissues and low accumulation of activity in organs with NaI-symporter expression. Both radiolabeled (HE)_3_-G3 variants had equal tumor uptake. Consequently, the indirect label provided higher tumor-to-blood and tumor-to-organ ratios compared with the direct label. Comparative Single Photon Emission Computed Tomography (SPECT)/CT imaging of HER2 expression in SKOV3 xenografts, using both radiolabeled DARPins, demonstrated the superior imaging contrast of the indirect label. Indirect radioiodination of (HE)_3_-G3 using SPIB could be further applied for SPECT and PET imaging with iodine-123 and iodine-124.

## 1. Introduction

The mechanism of action of targeted anti-cancer biopharmaceuticals includes the molecular recognition of gene products that are overexpressed or mutated in malignant tumors. These targeted biopharmaceuticals are successfully used for treatment of disseminated cancer. An overexpression of a molecular target is often a prerequisite for a tumor response to such treatment. Radionuclide molecular imaging of a target’s expression in tumors might be a facile way to stratify patients for treatment using biopharmaceuticals [1]. This approach is not prone to sampling errors and is non-invasive, which permits determining the expression level repeatedly in order to monitor its changes during disease progression [1]. During the last decade, new types of molecular imaging probes based on engineered scaffold proteins (ESPs) have emerged [2]. ESPs are usually selected by molecular display techniques. The presence of a rigid scaffold, which holds variable amino acids in a well-defined spatial structure, reduces the entropic penalty and permits selection of binders with very high (down to picomolar range) affinity for pre-determined molecular targets [3,4]. The small size of the ESP promotes rapid localization of ESP-based imaging probes in tumors. Since the molecular weight of ESPs (4–20 kDa) are below the cut-off values for glomerular filtration (ca. 60 kDa), unbound tracers are rapidly excreted via the kidneys. This results in a low background activity in the body and, consequently, a high contrast of imaging [2].

It is important to realize that the small size of an ESP (although necessary) is not a sufficient precondition for high-contrast molecular imaging. The labeling strategy (i.e., the selection of radionuclide and chelator or prosthetic group for its coupling to a tumor-seeking protein) and linker moiety play an essential role as well [5]. The coupling of an atom or a group of atoms modifies the surface of a targeting protein, changing its distribution of lipophilic patches, charge, and electron density. Accordingly, this may influence both its affinity to a molecular target and off-target interactions. Also, the fate of the radiometabolites might be different depending on the physico-chemical properties of a chelator or a prosthetic group/linker [5]. This can substantially affect the imaging contrast. Thus, systematic studies concerning the influence of labeling chemistry on the imaging properties of ESP-based radionuclide imaging probes are necessary to provide high sensitivity of imaging.

The scaffold of designed ankyrin repeat proteins (DARPins) is formed by modules consisting of a β-turn and two antiparallel α-helices [6,7]. DARPins may include from four to six of such repeats and their molecular weight can accordingly vary between 14 and 18 kDa. DARPin-mediated radionuclide imaging has focused thus far on the visualization of human epidermal growth factor receptor 2 (HER2) expression [8,9,10,11,12]. HER2 is a receptor tyrosine kinase that normally regulates cellular mitotic activity and apoptosis [13,14]. Overexpression of HER2 has been documented in several malignancies, including carcinomas of the breast, stomach, and esophagus [15,16,17]. The use of HER2-targeted therapeutics (e.g., monoclonal antibodies trastuzumab and pertuzumab, and a conjugate of trastuzumab with maytansine) results in a significant survival increase of breast, gastric and, gastroesophageal-junction cancer patients [17,18]. A high expression of HER2 is a strong predictive biomarker for successful treatment of such tumors, and an analysis of the HER2-expression level is mandatory for stratification of patients for therapy [19,20,21]. Therefore, the development of probes for HER2 imaging is an actively evolving research area in radiopharmaceutical science [22,23].

Molecular-display selection of two HER2-binding DARPins, 9_29 (phage display selection, *K_D_* = 3.8 nM) [24] and G3 (ribosome display selection, *K_D_* = 90 pM) [25], has been reported. The use of both radiometal (^99m^Tc and ^111^In) [8,9,10,12] and radioiodine labels [8,9,10,11] for these DARPins has been preclinically evaluated for imaging purposes. It should be noted that radiometal labels are usually residualizing, i.e., they become intracellularly trapped after internalization and proteolytic degradation of the targeting protein in lysosomes. The radiohalogen labels are typically non-residualizing, i.e., their radiometabolites can diffuse though lysosomal and cellular membranes and leak from cells. It has been found that the smaller high-affinity G3 DARPin provides better imaging than the bigger DARPin 9_29, irrespective of the label’s nature [10]. Furthermore, a (HE)_3_-G3 variant (having a hydrophilic HEHEHE-tag at its N-terminus) demonstrates the most favorable biodistribution profile, in particular having the lowest hepatic uptake [8,12]. The internalization of DARPins by HER2-expressing cancer cells is slow [9,10]. Therefore, the residualizing character of the label has had no pronounced effect on the tumor’s retention of activity. On the other hand, internalization of DARPins in excretory organs, kidneys and liver, is apparently rapid. The use of residualizing radiometal labels results in long retention of radionuclides, but the radiometabolites of a direct radioiodine label are cleared fast from these organs [8,9,10,11,12]. This results in higher tumor-to-organ ratios (imaging contrast) in the case of the radioiodine label. A high tumor-to-liver ratio is particularly important, since the liver is a frequent metastatic site for breast cancer. The issue with the direct radioiodine label is the re-distribution of radiometabolites, which results in elevated radionuclide uptake in Na/I-symporter-expressing organs and an elevated retention of activity in the blood [10]. Saturation of the *Na/I*-symporter with non-radioactive potassium iodide solves this problem to a certain extent. However, this result has not been quite reproducible and results in high uptakes in Na/I-symporter-expressing organs, e.g., elevated stomach uptake [10,11].

Previously, we have demonstrated that labeling of other ESPs (affibody molecules and Albumin-Binding Domain Derived Affinity Proteins (ADAPTs)) using [^125^I]I-iodo-((4-hydroxyphenyl)ethyl)maleimide (HPEM) results in rapid excretion of radiometabolites, and low uptake in the salivary glands and stomach [26,27]. However, application of this strategy for the labeling of anti-HER2 DARPin G3 resulted in elevated hepatobiliary excretion of the conjugate, causing decreased tumor uptake and a high activity level in the contents of the gastrointestinal tract [11]. This might complicate imaging of extrahepatic abdominal metastases.

An alternative approach to the introduction of a radioiodine label, with rapid excretion of its radiometabolites, is an indirect radioiodination using N-succinimidyl-*para*-iodobenzoate (SPIB) [28]. This method has been successfully applied for labeling monoclonal antibodies [28,29,30] and affibody molecules [30].

The aim of this study was to test the hypothesis that the use of N-succinimidyl-*para*-iodobenzoate results in radioiodinated DARPin G3 being able to preserve its strength and specificity of binding to HER2-expressing cells and provide high-contrast imaging of HER2-expressing tumors in vivo. To test this hypothesis, (HE)_3_-G3 with the most favorable biodistribution profile was selected. (HE)_3_-G3 was radioiodinated using N-succinimidyl-*para*-iodobenzoate (designated further as [^125^I]I-PIB-(HE)_3_-G3). The same DARPin variant, which was directly radioiodinated using Chloramine-T (designated further as [^125^I]I-(HE)_3_-G3), was used as a control. The affinity and specificity of [^125^I]I-PIB-(HE)_3_-G3 and [^125^I]I-(HE)_3_-G3’s binding to HER2-expressing cell lines in vitro, their processing by HER2-expressing cells, and in vivo targeting properties were all compared head-to-head.

## 2. Results

### 2.1. Radiolabeling and Stability

Two methods for the radioiodination of (HE)_3_-G3 DARPin were used: direct labeling using chloramine-T and indirect labeling using a N-succinimidyl-*para*-iodobenzoate (SPIB) labeling reagent.

Direct labeling was performed with a 98 ± 1% radiochemical yield and 87 ± 5% isolated yield. The maximum apparent specific activity for [^125^I]I-(HE)_3_-G3 was 1.4 MBq/µg (apparent molar activity 20.0 GBq/µmol). Indirect labeling was performed with a 15 ± 1% radiochemical yield and 13 ± 1% isolated yield. The maximum apparent specific activity for [^125^I]I-PIB-(HE)_3_-G3 was 0.3 MBq/µg (apparent molar activity 4.3 GBq/µmol). Purification using size-exclusion chromatography provided both radiolabeled proteins with radiochemical purities over 98%. Both direct and indirect labeling methods provided stable attachment of the radioiodine to (HE)_3_-G3 DARPin (Table 1).

### 2.2. In Vitro Characterization of Radiolabeled DARPins

**Figure 1:**
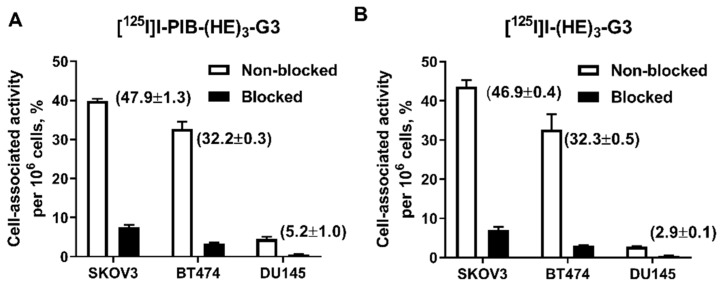
Binding specificity of: (**A**) [^125^I]I-PIB-(HE)_3_-G3 and (**B**) [^125^I]I-(HE)_3_-G3 to HER2-expressing SKOV3, BT474 and DU145 cells. In the blocked group, receptors were saturated with a 100-fold excess of non-labeled (HE)_3_-G3. Numbers in parentheses show the percentage of cell-bound activity per whole sample, i.e., without correction per cell number. Data are presented as the mean of three samples ± standard deviation (SD).

The binding specificity of [^125^I]I-PIB-(HE)_3_-G3 and [^125^I]I-(HE)_3_-G3 to HER2 was studied using HER2-expressing SKOV3, BT474 and DU145 cells. Pre-saturation of HER2 receptors by a large excess of non-labeled (HE)_3_-G3 resulted in a significant (*p* < 0.001) decrease in binding of both radiolabeled DARPins to the cells (Figure 1). The level of cell-bound activity was dependent on the level of HER2 expression and was higher for SKOV3 (1.6 × 10^6^ receptors/cell [31]) and BT474 (1.2 × 10^6^ receptors/cell [32]) cells in comparison with DU145 cells (5 × 10^4^ receptors/cell [33]).

The kinetics of [^125^I]I-PIB-(HE)_3_-G3 and [^125^I]I-(HE)_3_-G3’s binding to HER2-expressing SKOV3 cells was measured using the LigandTracer. The binding was fitted to a 1:2 interaction model, as previously reported for the G3-H_6_ DARPin [10]. A high affinity interaction in the picomolar range, and a low affinity interaction in the nanomolar range were observed for both labeled proteins, with their values being similar to the G3-H_6_ DARPin (Table 2). This confirmed that both direct and indirect labeling methods provided radiolabeled (HE)_3_-G3 proteins that retained their binding characteristics to HER2.

**Figure 2:**
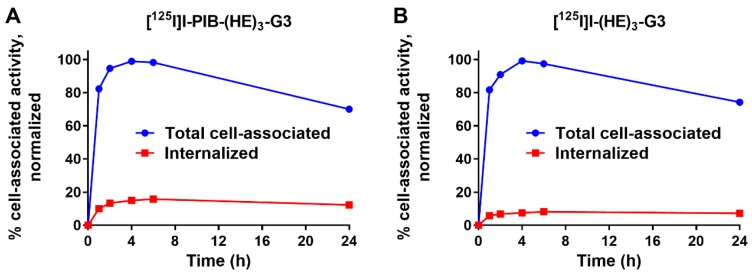
Cellular processing of: (**A**) [^125^I]I-PIB-(HE)_3_-G3 and (**B**) [^125^I]I-(HE)_3_-G3 in HER2-expressing SKOV3 cells during continuous incubation at 37 °C. Data are presented as the mean of three samples ± SD. Error bars may not be visible if they are smaller than symbols.

Processing of [^125^I]I-PIB-(HE)_3_-G3 and [^125^I]I-(HE)_3_-G3 by HER2-expressing SKOV3 cells was studied during continuous incubation for 24 h (Figure 2). The general pattern of cellular processing was typical for a non-residualizing label. After the maximal accumulation at 4 h, a slow decrease in cell-associated activity was observed due to the diffusion of radiocatabolites from the cells. The internalized fraction for PIB labeled [^125^I]I-PIB-(HE)_3_-G3 was ca. two times higher compared to that of directly labeled [^125^I]I-(HE)_3_-G3 at all time points.

### 2.3. Animal Studies

**Figure 3:**
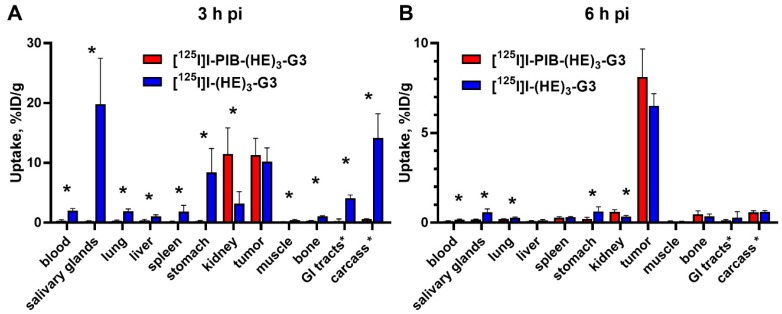
Comparative biodistribution of [^125^I]I-PIB-(HE)_3_-G3 and [^125^I]I-(HE)_3_-G3 at (**A**) 3 h and (**B**) 6 h pi in BALB/C nu/nu mice bearing HER2-expressing SKOV3 xenografts. Data are presented as the mean ± SD from four mice. An asterisk marks a significant difference between values, * *p* < 0.05; unpaired *t*-test. Data for intestines with content and carcass are presented as %ID per whole sample.

A comparison of biodistribution and tumor targeting properties between [^125^I]I-PIB-(HE)_3_-G3 and [^125^I]I-(HE)_3_-G3 was performed in BALB/C nu/nu mice, bearing HER2-expressing SKOV3 xenografts, at 3 and 6 h post-injection (pi) (Figure 3). At 3 h pi, both radiolabeled DARPins showed rapid clearance from the blood, low retention in excretory organs, and provided similar levels of tumor uptake (*p* > 0.05; unpaired *t*-test). However, the retention of activity in the blood and all tissues and organs (except the kidneys) was much higher for directly labeled [^125^I]I-(HE)_3_-G3 (*p* <0.05; unpaired *t*-test). Notably, in the case of the indirectly labeled DARPin the activity was efficiently cleared from the body already at 3 h pi. The indirect label provided a major decrease in the accumulation of iodine-125 radiocatabolites in organs with expression of Na/I-symporters (ca. 80 times reduction in the salivary glands and ca. 30 times reduction in the stomach). At 6 h pi a further decrease in activity due to clearance from normal organs and tissues was observed for both labels, with tumors having the highest activity uptake.

**Figure 4:**
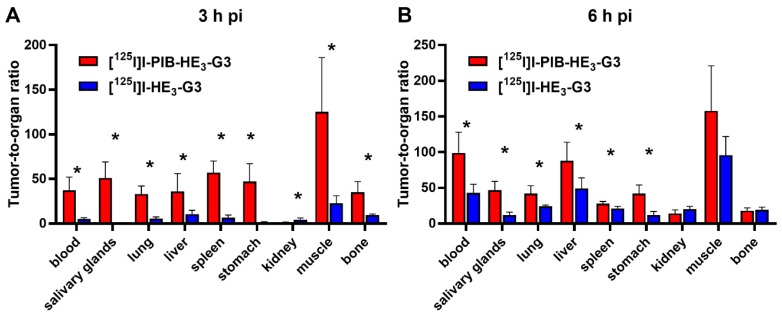
Comparison of tumor-to-organ ratios for [^125^I]I-PIB-(HE)_3_-G3 and [^125^I]I-(HE)_3_-G3 at (**A**) 3 h and (**B**) 6 h pi in BALB/C nu/nu mice bearing HER2-expressing SKOV3 xenografts. Data are presented as the mean ± SD from four mice. An asterisk marks a significant difference between values, * *p* < 0.05.

The tumor-to-organ ratios for the indirectly labeled [^125^I]I-PIB-(HE)_3_-G3 were significantly (*p* < 0.05) higher for all organs and tissues, except the kidneys, at 3 h pi and for the majority of organs at 6 h pi in comparison with the directly labeled DARPin (Figure 4).

**Figure 5:**
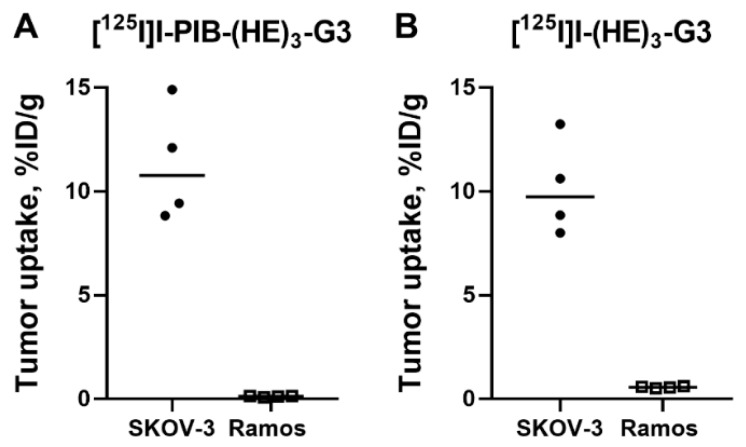
Specificity of tumor targeting by: (**A**) [^125^I]I-PIB-(HE)_3_-G3 and (**B**) [^125^I]I-(HE)_3_-G3 at 3 h pi in HER2-expressing SKOV3 and HER2-negative Ramos xenografts. Data are presented as individual data points from four mice.

The specificity of HER2 targeting by the radioiodinated DARPins was confirmed in BALB/C nu/nu mice bearing HER2-negative Ramos xenografts at 3 h pi (Figure 5). The tumor uptake of [^125^I]I-PIB-(HE)_3_-G3 and [^125^I]I-(HE)_3_-G3 was significantly (*p* < 0.0005; unpaired *t*-test) lower in Ramos xenografts than in SKOV3 xenografts.

**Figure 6:**
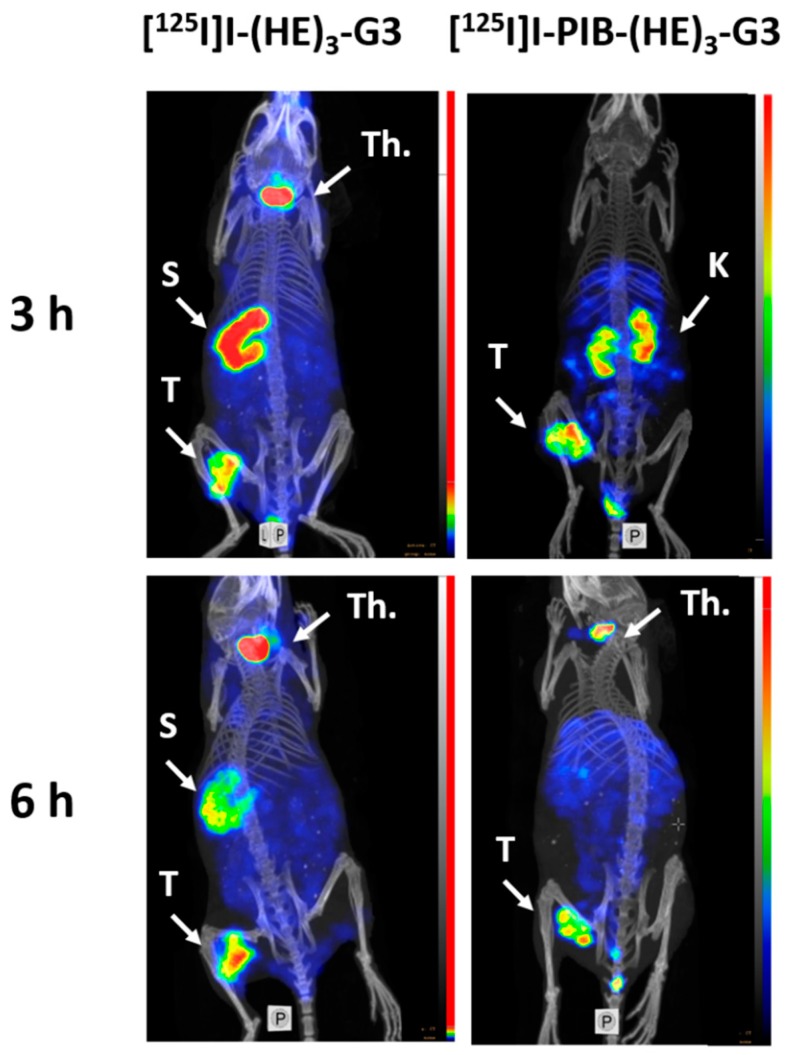
MicroSPECT/CT imaging of HER2 expression in BALB/C nu/nu mice bearing HER2-expressing SKOV3 xenografts at three and 6 h pi using [^125^I]I-(HE)_3_-G3 and [^125^I]I-PIB-(HE)_3_-G3. S = stomach, T = tumor, K = kidneys, and Th = thyroid. Before imaging, the mice were euthanized and their urinary bladders were excised.

**Figure 7:**
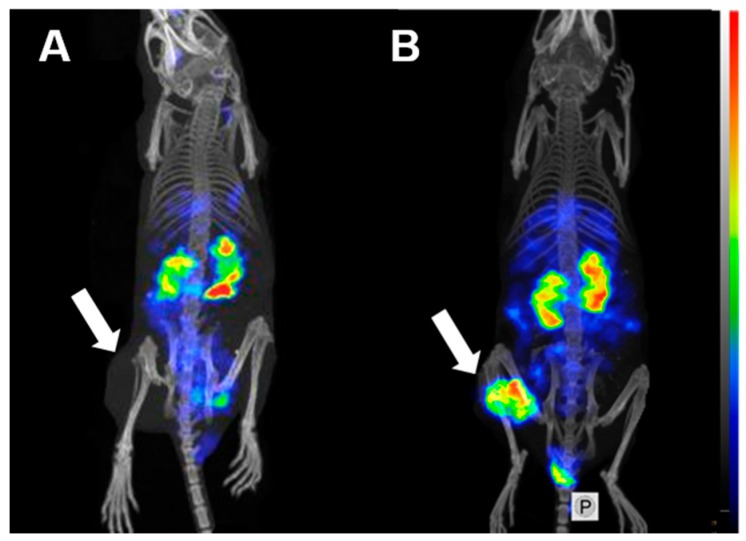
MicroSPECT/CT imaging of HER2 expression in BALB/C nu/nu mice bearing: (**A**) HER2-negative Ramos xenografts (negative control) and (**B**) HER2-positive SKOV3 xenografts at 3 h pi using [^125^I]I-PIB-(HE)_3_-G3. Arrows point at tumors. Before imaging, the mice were euthanized and their urinary bladders were excised.

Single Photon Emission Computed Tomography (SPECT)/CT imaging confirmed the results of the ex vivo biodistribution measurements. Both radiolabeled variants, [^125^I]I-PIB-(HE)_3_-G3 and [^125^I]I-(HE)_3_-G3, clearly visualized HER2-expressing SKOV3 xenografts (Figure 6). Appreciable accumulation of activity in organs with Na/I-symporter expression (salivary glands and stomach) was detected for directly labeled [^125^I]I-(HE)_3_-G3. The best performing variant, [^125^I]I-PIB-(HE)_3_-G3, showed no tumor uptake in HER2-negative Ramos xenografts (Figure 7), which confirmed the specificity of tumor targeting.

## 3. Discussion

In this study, we used ^125^I (T_1/2_ = 59.4 d) as a convenient surrogate for the radionuclides ^123^I (T_1/2_ = 13.3 h) and ^124^I (T_1/2_ = 4.2 d), which can be used for imaging using SPECT and PET, respectively. We have shown that indirect labeling using N-succinimidyl-*para*-iodobenzoate provides a stable covalent attachment of radioiodine to (HE)_3_-G3 (Table 1). The challenge reaction, run with a large excess of non-radioactive NaI, excluded the probability of any unstable attachment of unreacted [^125^I]iodide to the positively charged side-chains of amino acids in (HE)_3_-G3. 

One possible issue with the selected labeling approach was the coupling of the [^125^I]I-PIB label to the amino groups of lysine residues. Lys^43^ is essential for binding to HER2, while Lys^68^ and Lys^101^ are located in the binding site close to randomized amino acids [25]. Obviously, modification of these lysines might have interfered with the high-affinity binding to HER2. However, in vitro experiments demonstrated that the specificity of [^125^I]I-PIB-(HE)_3_-G3’s binding to HER2-expressing cells was well preserved, and the binding level correlated with the expression of HER2 in the tested cell lines (Figure 1). Furthermore, the affinity values for [^125^I]I-PIB-(HE)_3_-G3 and [^125^I]I-(HE)_3_-G3’s binding to living HER2-expressing cells were quite close, which indicated an unaltered binding strength (Table 2). Interestingly, the affinity measurements suggested that there were two types of interactions occurring with HER2 on cellular membranes, one with picomolar and the other with nanomolar affinity. Such dual interactions are a characteristic feature of DARPins [9,10] as well as other types of binders, e.g., affibody molecules, [34], ADAPTs [27], and monoclonal antibodies [34] specific to HER2. A possible explanation could be that the binding strength to the monomeric and dimeric forms, of HER2 receptors on the cellular membrane, might be different. It should be noted that the radiochemical yield was noticeably lower compared to data from other reports concerning the use of N-succinimidyl-*para*-iodobenzoate (see, e.g., [26]). This was not expected as the sequence of G3 contains eight lysine residues. However, it might be that not all these lysines are easily accessible for interaction with N-succinimidyl-*para*-iodobenzoate. 

The cellular processing pattern (Figure 2) was similar for [^125^I]I-PIB-(HE)_3_-G3 and [^125^I]I-(HE)_3_-G3: the rapid increase of total cell-bound activity up to 4 h incubation was followed by a slow decrease. This is typical for slowly internalizing HER2 binders with non-residualizing labels [10]. In the case of rapid internalization of a radiolabeled ligand, the cellular uptake of a non-residualizing label peaks much earlier and the release of cell-associated radioactivity is appreciably faster [35]. In the case of a residualizing label, a plateau or a continuous growth of a cell-associated activity is typically observed depending on the rates of internalization and de novo production of receptors [10,27,36]. The level of internalized activity was low, which is natural for a “leaky” non-residualizing label. However, the internalized activity for [^125^I]I-PIB-(HE)_3_-G3 was somewhat higher than for [^125^I]I-(HE)_3_-G3. The internalized fraction is determined by several processes: internalization of a receptor-ligand complex, its trafficking to lysosomal compartments, proteolytic degradation and the “leakage” of radiometabolites. It is not likely that the internalization of [^125^I]I-PIB-(HE)_3_-G3 is faster than that of [^125^I]I-(HE)_3_-G3. Quite likely, the rates of intracellular trafficking of internalized conjugates and proteolysis are similar for both conjugates. Thus, the observed phenomenon might be explained by the slower escape of [^125^I]I-PIB-(HE)_3_-G3 metabolites through lysosomal and cellular membranes.

In vivo, the uptake of both [^125^I]I-PIB-(HE)_3_-G3 and [^125^I]I-(HE)_3_-G3 was significantly (*p* < 0.0005) higher in HER2-expressing SKOV3 compared to HER2-negative Ramos xenografts (Figure 5). This clearly demonstrated that the tumor accumulation was HER2-mediated. The low level of uptake in HER2-negative tumors suggested that unspecific tumor uptake, due to the enhanced permeability and retention (EPR) effect, is negligible for radioiodinated DARPins. Therefore, the risk of a false-positive finding is very low. This contrasts with the substantial unspecific uptake of anti-HER2 antibodies, which might be in the range of 20 to 50%, depending on the specific one used [32,37].

The uptake of [^125^I]I-PIB-(HE)_3_-G3 and [^125^I]I-(HE)_3_-G3 in HER2-positive xenografts did not differ significantly (Figure 3) at both time points. This was quite expected since the size of both DARPins was approximately the same, and the size determines the rates of extravasation and diffusion in the extracellular space of tumors. The affinities of both variants to HER2 were also approximately equal, and there was no appreciable difference in their cellular processing. Thus, the major factors determining the localization of activity in tumors had a similar impact on [^125^I]I-PIB-(HE)_3_-G3 and [^125^I]I-(HE)_3_-G3. There was, however, a striking difference in activity uptake in normal tissues. The uptake in the majority of tissues was much higher for [^125^I]I-(HE)_3_-G3 at 3 h after injection (Figure 3). Even though this difference had decreased 6 h after injection, uptake in the blood, salivary glands, stomach and gastrointestinal tract was still significantly higher in the case of direct radioiodination (Figure 3). The most reasonable explanation for such disparity of activity distribution is the difference in re-distribution of radiometabolites. Indeed, evaluation of G3 DARPins with residualizing ^111^In- and ^99m^Tc-labels demonstrated a very high degree of re-absorption in the kidneys (50–60% of injected activity per whole organ) [8,10,11]. This high reabsorption was independent of the label position as well as the chemical nature of the nuclide and chelator. Very likely, the same degree of re-absorption takes places in the case of radioiodinated G3. However, the internalization of re-absorbed peptides in the proximal tubuli of kidneys is rapid [38], and the radiometabolites from non-residualizing radioiodine labels diffuse from the cells. Part of these metabolites might be released into urine, but a part is returned back to the blood circulation. The main intracellular radiometabolites of the direct radioiodine label are iodide and iodotyrosine [35], which are taken up by the system of enzymes and transporters involved in the handling of thyroid hormones. This leads to an elevated retention of the radioiodine in the body. On the contrary, the main radiometabolites of PIB labels, iodobenzoate-lysine adducts, are rapidly excreted from the body without any interactions. An interesting exception from this pattern was the significantly higher renal activity retention of [^125^I]I-PIB-(HE)_3_-G3 compared with [^125^I]I-(HE)_3_-G3 (Figure 3). This might correlate with the somewhat elevated internalized fraction in the cellular processing study and reflect, to a some extent, stronger residualizing properties of the [^125^I]I-PIB-label.

The lower retention of activity in normal organs resulted in appreciably higher tumor-to-organ ratios for [^125^I]I-PIB-(HE)_3_-G3 (Figure 4). High tumor-to-organ ratios have a paramount value as they determine the imaging contrast and therefore the sensitivity of radionuclide diagnostics. As high as possible contrast is essential for visualization of small metastases with a diameter comparable or smaller than the spatial resolution of an imaging device [39]. Particularly important, is the tumor-to-liver ratio because hepatic metastases are common in both breast and gastric cancer [40,41]. The use of [^125^I]I-PIB-(HE)_3_-G3 provides a tumor-to-liver ratio of 88 ± 26 at 6 h after injection. This is nearly two times higher than the ratio for [^125^I]I-(HE)3-G3 at the same time point (49 ± 15). It is also substantially higher than the best tumor-to-liver ratios provided by other HER2-targeting DARPins, such as [^111^In]In-(HE)_3_-G3 (12.0 ± 3.6 at 24 h after injection) [8], or [^99m^Tc]Tc(CO)_3_-(HE)_3_-G3 (5.9 ± 0.9 at 24 h after injection) [12].

Experimental SPECT/CT imaging (Figure 6 and Figure 7) confirmed the results of the ex vivo measurements, i.e., the specific character of [^125^I]I-PIB-(HE)_3_-G3 accumulation in HER2 expressing xenografts and appreciably better imaging contrast in comparison with [^125^I]I-(HE)_3_-G3.

The use of N-succinimidyl-*para*-iodobenzoate for labeling (HE)_3_-G3 provided clear advantages in comparison to the use of iodo-[(4-hydroxyphenyl)ethyl]maleimide. In the case of [^125^I]I-HPEM-G3-GGGC [11], there was an apparent shift toward hepatic uptake and hepatobiliary excretion of activity compared to the biodistribution of its directly radioiodinated counterpart. At 4 h after injection, the hepatic uptake of [^125^I]I-HPEM-G3-GGGC was 1.6 ± 0.5%ID/g, and the activity in the contents of the gastrointestinal tract was 22 ± 4%ID. In this study, the hepatic uptake of [^125^I]I-PIB-(HE)_3_-G3 was much lower, 0.4 ± 0.2 and 0.10 ± 0.02%ID/g, at 3 and 6 h after injection, respectively. The average activity in the contents of the gastrointestinal tract was below 0.5%ID. Interestingly, the use of iodo-[(4-hydroxyphenyl)ethyl]maleimide for labeling of affibody molecules also caused elevated hepatic uptake compared to the use of N-succinimidyl-*para*-iodobenzoate [26], but this difference was not so critical due to higher tumor uptake. It is possible that the hepatic uptake is dependent on the lipophilic terminal-modifications of short protein

It has to be noted that selecting the optimal labeling chemistry depends on cellular processing of the imaging agent after it has bound to the corresponding molecular target. An important factor for having selected a non-residualizing label in this study was the information that the internalization rate of G3 derivatives is low. Experiments with residualizing ^99m^Tc-labels demonstrate that the internalized fraction is less than 15% after 8 h [10,12]. This is not always the case. For example, 5F7GGC Nanobody is internalized very rapidly after binding to HER2-expressing cells. The internalized fraction is as much as 50–70% after only one hour [42,43]. Accordingly, the selection of residualizing labels, such as N(ɛ)-(3-[¹³¹I]iodobenzoyl)-Lys⁵-N(α)-maleimido-Gly¹-GEEEK ([¹³¹I]IB-Mal-D-GEEEK) [42], or N-succinimidyl 4-guanidinomethyl 3-[^125/131^]I-iodobenzoate (*I-SGMIB) [43], was a logical decision. The cell studies demonstrated appreciably better cellular retention of activity when residualizing radioiodine labels were used [42,43]. In both cases, the use of residualizing labels resulted in appreciably better targeting properties. Particularly successful was the use of *I-SGMIB, which provided not only the highest tumor uptake but also the fastest clearance from normal organs. Thus, the molecular design of an imaging probe should be based on careful biological studies.

## 4. Materials and Methods

Production and characterization of DARPin (HE)_3_-G3 with its histidine-glutamate tag at the N-terminus was previously described in [12]. Iodine [^125^I]NaI was purchased from Perkin Elmer Sverige AB (Sweden). Purification of radiolabeled proteins was performed using NAP-5 size-exclusion columns (GE Healthcare, Uppsala, Sweden). Instant thin-layer chromatography (iTLC) analysis was performed using iTLC silica gel-impregnated chromatography paper (Varian, Lake Forest, CA, USA) in water:acetone (1:4) as a mobile phase. The activity in samples was measured using an automated gamma-spectrometer with a NaI(TI) detector (1480 Wizard, Wallac, Helsinki, Finland). HER2-expressing SKOV3, BT474 and DU145 cells and HER2-negative Ramos cells from the American Type Culture Collection (ATCC) were cultured in RPMI medium supplemented with 10% fetal bovine serum (Merck, Darmstadt, Germany), 2 mM L-glutamine, 100 IU/mL penicillin and 100 µg/mL streptomycin in a humidified incubator with 5% CO_2_ at 37 °C, unless stated otherwise. The unpaired two-tailed t-test was used to determine statistical significance (*p* < 0.05) for in vitro and in vivo specificity experiments (GraphPad Software, San Diego, CA, USA). Comparison of biodistribution data for every time point was performed using the unpaired two-tailed t-test (GraphPad Software, San Diego, CA, USA). Animal studies were approved by the Ethics Committee for Animal Research in Uppsala, Sweden (decision C5/16, 26 February 2016) and performed according to the national legislation on protection of laboratory animals.

### 4.1. Direct Radioiodination

Direct labeling of DARPin (HE)_3_-G3 with iodine-125 was performed using the chloramine-T method as described previously [10].

### 4.2. Indirect Radioiodination

Indirect labeling of (HE)_3_-G3 was performed in two steps following previously described methods [29,30]. First, an aqueous solution of 0.1% acetic acid (5 µL) was added to the solution of radioiodine (5 µL, 13 MBq). Then, N-succinimidyl-p-(trimethylstannyl)benzoate (6.5 nmoles, 2.5 µg, 2.5 µL of 1 mg/mL in 5% acetic acid in methanol) was added. Iodination was started by adding chloramine-T (40 µg, 10 µL, 4 mg/mL in water), the solution was incubated for five min at room temperature. The reaction was stopped by addition of sodium metabisulfite (60 µg, 10 µL, 6 mg/mL in water). Then, DARPin (HE)_3_-G3 (4.1 nmoles, 60 µg, 7.7 µL of 8.2 mg/mL in PBS) in 70 µL of 0.07 M borate buffer (pH 9.3) was added. After 30 min of incubation at room temperature, the radiolabeled conjugate was purified on a NAP-5 column and eluted with PBS.

### 4.3. Label Stability

The radiolabel stability was tested *in vitro* by incubating [^125^I]I-PIB-(HE)_3_-G3 and [^125^I]I-(HE)_3_-G3 with a 5000-fold molar excess of KI in PBS at room temperature for 3 h. Control samples were incubated in PBS. Samples were analyzed by iTLC.

### 4.4. In Vitro Characterization

The binding kinetics of the radiolabeled DARPins [^125^I]I-PIB-(HE)_3_-G3 and [^125^I]I-(HE)_3_-G3 to living SKOV3 cells was measured using the LigandTracer (Ridgeview Instruments AB, Vänge, Sweden) as described previously [44]. Kinetics of binding to and dissociation from cells were recorded at room temperature in real time. Association was measured by adding increasing concentrations of radiolabeled DARPins (0.5 and 2 nM) to cells. TraceDrawer Software (version 1.7.1; Ridgeview Instruments AB, Sweden) was used to calculate the dissociation constants based on the association and dissociation rates.

The binding specificity of both radiolabeled DARPins was studied using HER2-expressing SKOV3, BT474 and DU145 cells as described previously [11]. Cells were seeded in 3 cm petri dishes (ca. 10^6^ cells per dish) one day before the experiment, a set of three dishes was used for each group. The evaluation of both DARPins was performed side-by-side using cells from the same passage. Two sets of dishes per cell line were used. In one set, HER2 receptors were pre-saturated with 100 nM of non-radiolabeled (HE)_3_-G3 for 30 min. Then the cells in both sets were incubated with 1 nM of the corresponding labeled DARPin for one h at 37 °C. Afterwards, the media was collected, cells were washed with serum-free media, and trypsin was added to collect the cells. The average cell number per dish was counted. The activity in the samples was measured. Cell-bound activity was calculated per 10^6^ cells.

Cellular processing of radiolabeled DARPins by SKOV3 cells was studied during continuous incubation using an acid-wash method [45]. The cells were seeded (ca. 10^6^ cells per dish) in three dishes for each time point. The radiolabeled DARPins (1 nM) were added to the cells and incubated at 37 °C. At one, two, four, six and 24 h after addition, the media was collected from one set of dishes and the cells were washed with serum-free media. To collect the membrane-bound DARPins, the cells were treated with a buffer containing 0.2 M glycine and 4 M urea (pH 2.0) on ice for five min, the buffer was collected, and the cells were washed once with the same buffer. To collect the internalized fraction, the cells were treated with 1 M NaOH for 30 min and the lysates were collected. The activity in every fraction was measured. The cell-bound activity was calculated and the maximum value in each dataset (individually for [^125^I]I-PIB-(HE)_3_-G3 and for [^125^I]I-(HE)_3_-G3) was taken as 100% and the data were normalized to that value.

### 4.5. Biodistribution Studies

Female Balb/c nu/nu mice were subcutaneously implanted with SKOV3 cells (8 × 10^6^ per mouse) four weeks before experiments or with Ramos cells (10 × 10^6^ per mouse) three weeks before experiments. At the time of experiments, the average mouse weight was 19.1 ± 0.7 g, the average tumor weight in the SKOV3 group was 0.30 ± 0.13 g and average tumor weight in the Ramos group was 0.38 ± 0.17 g.

A group of five mice with SKOV3 xenografts or a group of four mice with Ramos xenografts was injected intravenously with 3.2 µg of a radiolabeled DARPin (20 kBq, in 100 µL 1% BSA in PBS). At three and 6 h pi., mice were injected intraperitoneally (i.p.) with ketamine and xylazine solution (250 mg/kg of ketamine, 25 mg/kg xylazine) and sacrificed by heart puncture. The blood, organs and tumors were collected, weighed, and their activity was measured. The percentage of injected dose per gram of sample (%ID/g) was calculated, except for the intestines with contents and carcass, where it was calculated as %ID per whole sample.

SPECT/CT imaging was performed to obtain visual confirmation of the ex vivo biodistribution studies. Two mice bearing SKOV3 xenografts with high HER2 expression were injected with [^125^I]I-PIB-(HE)_3_-G3 (8 µg, 2.9 MBq, 100 µL 1% BSA in PBS) and two mice were injected with [^125^I]I-(HE)_3_-G3 (8 µg; 7.4 MBq, 100 µL 1% BSA in PBS), imaging was performed at 3 and 6 h pi. One mouse bearing a Ramos xenograft was injected with [^125^I]I-PIB-(HE)_3_-G3 (8 µg; 1.2 MBq, 100 µL 1% BSA in PBS), and imaging was performed at 3 h p.i. Mice were euthanized by i.p. injection of ketamine and xylazine solution (500 mg/kg of ketamine, 50 mg/kg xylazine), urinary bladders were removed. SPECT/CT imaging was performed using nanoScan SPECT/CT (Mediso Medical Imaging Systems Ltd., Budapest, Hungary). The acquisition time was 20 min. CT scans were acquired using the following parameters: X-ray energy peak of 50 keV; 670 µA; 480 projections; and 5.26 min acquisition time. SPECT raw data were reconstructed using Tera-Tomo™ 3D SPECT reconstruction technology (version 3.00.020.000; Mediso Medical Imaging Systems Ltd.): normal dynamic range; 30 iterations; and one subset. CT data were reconstructed using Filter Back Projection in Nucline 2.03 Software (Mediso Medical Imaging Systems Ltd.). SPECT and CT files were fused using Nucline 2.03 Software and are presented as maximum intensity projections (MIP) in the RGB color scale.

## 5. Conclusions

Radioiodination of the anti-HER2 DARPin (HE)_3_-G3 using N-succinimidyl-*para*-iodobenzoate provides superior contrast compared to direct radioiodination. After slight re-optimization of the labeling chemistry, this method might be used for imaging of HER2 expression in tumors using ^123^I (SPECT) and ^124^I (PET). This study demonstrates the importance of labeling chemistry in the development of probes for radionuclide molecular imaging.

## Figures and Tables

**Table 1:** In vitro stability of [^125^I]I-PIB-(HE)_3_-G3 and [^125^I]I-(HE)_3_-G3. Samples were incubated with 5000-fold molar excess of KI or in PBS (control) at room temperature for 3 h. Analysis was performed in duplicates. Data for each labeled protein was normalized to its initial radiochemical purity, taken as 100%.

Test Solution	Normalized DARPin-Associated Activity, %	
	[^125^I]I-PIB-(HE)_3_-G3	[^125^I]I-(HE)_3_-G3
PBS (control)	100 ± 0	99 ± 1
5000-fold KI	99 ± 0	100 ± 0

**Table 2:** Equilibrium dissociation constants for the high affinity (*K_D1_*) and lower affinity (*K_D2_*) interactions between [^125^I]I-PIB-(HE)_3_-G3 or [^125^I]I-(HE)_3_-G3 and HER2-expressing SKOV3 cells. The assay was performed in duplicates.

	*K_D1_*(pM)	Weight (%)	*K_D2_*(nM)	Weight (%)
[^125^I]I-PIB-(HE)_3_-G3	54 ± 3	65	3.39 ± 0.02	35
[^125^I]I-(HE)_3_-G3	81 ± 4	60	2.76 ± 0.08	40

## Abbreviations

ADAPT	Albumin-Binding Domain Derived Affinity Protein
BSA	Bovine Serum Albumin
CT	Computed Tomography
DARPin	Designed Ankyrin Repeat Protein
HER2	Human Epidermal Growth Factor Receptor 2
iTLC	Instant Thin Layer Chromatography
MIP	Maximum Intensity Projections
PBS	Phosphate Buffered Saline
PET	Positron Emission Tomography
PIB	Para-Iodobenzoate
RGB	Red, Green and Blue Color Scale
SPECT	Single Photon Emission Computed Tomography
